# Hospital and Institutional News

**Published:** 1914-12-12

**Authors:** 


### HOSPITAL AND INSTITUTIONAL NEWS.

### A HOUSE GOVERNOR'S ELECTION.

Last Saturday the decision of the selection committee among the candidates for the post of house governor and secretary to the General Hospital, Birmingham, was made known. The following is the short list and the order in which they were interviewed by the selection committee: Mr. R. J. Coles, Addenbrooke's Hospital, Cambridge; Mr. Arthur Moore, Lincoln County Hospital; Mr. H. J. Dafforne, Ancoats Hospital, Manchester; Mr. A. H. Leaney, secretary of the Birmingham Municipal School of Art; Mr. Fred Smith, Warneford Hospital, Leamington; and Mr. Walter Pearson, Gravesend Hospital. The successful candidate is the local one, Mr. Alfred H. Leaney. Amongst selected candidates in the majority of cases an element of surprise attaches to the decision of men acting in groups; there is an incalculable factor, which can be appreciated only by the group's members, since they alone are acquainted with many things which cannot be apparent to the world outside. Hence the sympathy which is being felt for Mr. Coles is a natural one; for from the point of view of these outside observers he was described on the whole as the strongest candidate. Hospital experience was held to be essential by the Birmingham authorities, and the list of selected candidates denotes a careful selection with knowledge.

### THE NEW HOUSE GOVERNOR OF THE BIRMINGHAM GENERAL HOSPITAL.

Mr. Alfred H. Leaney, the new house governor, will receive every congratulation. He has a magnificent opportunity and a great responsibility in Birmingham, and no active spirit can desire more. Mr. Leaney had had previous hospital experience in London, where at one time he was a clerk in the Clerk's office at St. Bartholomew's Hospital, which he left soon after Mr. Hayes was appointed. He entered the Clerk's office in July 1901, and he remained there until December 1905, when he went to Birmingham as assistant secretary to the Birmingham Municipal School of Art, of which he subsequently became the secretary. During his term of office at St. Bartholomew's Mr. Leaney impressed his seniors with his general ability, which was felt to be far above the average, apart from his proficiency as a clerk in the mechanical arts of shorthand and typewriting. He acquired at St. Bartholomew's a reputation for efficiency and promise, together with a practical experience of institutional business and affairs. In a special testimonial, dated November 10, 1914, Mr. W. H. Cross states: "In the course of my long tenure, nearly forty years, of the office of Clerk, Mr. Leaney was one of the most capable of my assistants. He served under me for four and a half years, and had the opportunity, of which he fully and eagerly availed himself, of becoming acquainted with all branches of hospital administration." Mr. Cross kept himself informed of Mr. Leaney's career, and adds: "I venture to express an opinion that both his knowledge of hospital work and his subsequent general experience make him peculiarly fitted for the office" (*i.e.* as successor to the late Mr. Collins). Mr. Leaney evidently possesses many of the requisite qualifications for high office, and we congratulate him upon his appointment.

### A HOSPITAL THAT RAN AWAY.

If there is one domestic feature of the war in this country which is more regrettable than another, it is undoubtedly the reckles multiplication of subscription lists for all sorts of objects which private individuals wish to further. This mischievous epidemic of individual effort to run individual hobbies with a war-fund label in the name of charity might usefully engage the attention of the Censor. We hoped the committee of every voluntary hospital which tends the wounded would appreciate the fact that they have at present a ground of appeal that gives them the first claim upon all monetary gifts, as the public recognise. A communication we have received from Portsmouth, however, shows that there the hospital committee have committed the happy dispatch by bolting from their entrenchments as the first claimant who is completely entitled to support, on the appearance of an army of local war funds' promoters. Could ineptitude and ill-judgment join hands with less reason and more discredit to the hospital authorities than the Portsmouth position reveals? We are afraid the effect will be to injure permanently the financial revenues of the Royal Portsmouth Hospital. Is there not sufficient public and press opinion in Portsmouth to arouse those responsible to a sense of their duty as trustees for the wounded and the sick of this great military and naval centre?

### ANOTHER SECRETARY ON ACTIVE SERVICE.

Captain M. J. Prentice, who for the past four years has held the post of secretary to the Worthing Hospital, has tendered his resignation on account of having been placed in command of the local battalion of the National Reserves which is quartered in Kent. At a recent meeting of the committee a resolution—which has since been embodied in an illuminated address—was passed accepting with the greatest regret Captain Prentice's resignation, and testifying to his efficient and devoted work for the institution.

### FLASHLIGHTS IN OUR WAR NEWS PARAGRAPHS.

Attentive readers of these descriptions which, thanks to the co-operation of our special correspondents, we are enabled every week to publish (thus giving a bird's-eye view of the work which the hospitals are doing for the Allies in no other way obtainable), will have noticed the emergence of certain common facts. For instance, the wounded soldiers' preference for the voluntary hospital has been from time to time insisted on, while this week we observe that another general fact of interest, likely otherwise to have been missed, is the nature of the Channel crossing at the present time. For one correspondent points out the extremely trying nature of the sea-passage for the wounded during the prevalence of the late autumn gales. Another brings to the front a vivid picture of the Channel passage itself. This writer observes that not only was the passage of a lately arrived convoy distressing and laborious from the weather conditions, but also that it was specially lengthy owing to the necessity for avoiding mines and for taking the necessarily circuitous route now known only to pilots especially instructed for this work. We venture to think that a study of these paragraphs, dealing with every type of institution, for an effort has been made to give fair treatment to every institution irrespective of its size (even at the risk of disappointing those correspondents whose institutions from their size and importance naturally provided almost unlimited material), brings out in an unrivalled way the conditions under which the wounded are reaching us from base hospitals in France, to ship, from landing to train, from train to ambulance, and from ambulance to bed. On this, alas! long and tortuous journey lights are flashed from week to week, now at one point and now at another; and we remind every institution that each account is treated on its merits, and that a monopoly of space is not allowed to the largest or the busiest institutions. The small ones are also needed if proper perspective is to be kept.

### PRIVATES WELL TREATED IN GERMAN HOSPITALS.

We may note among the letters published from the Front in the lay Press one of interest to institutional readers for the testimony which it bears to the kind treatment extended, so far as the writer's private experience goes, to the wounded prisoners in German hospitals. "I am getting," says the passage in the letter relating to his life in hospital, "wholesome food and plenty of it. Do not worry about me. I am being very kindly treated, and what more can I want? When I leave hospital I may not get the chance of writing to you, so don't expect a card." It is interesting also to learn that the writer is described as being one of those who was taken prisoner during the London Scottish charge at Messines. It is to be hoped and believed that this statement is not only "uninspired" and correct, but also represents a general condition of well-being for our wounded in German institutions. So far as we are aware, no general complaints have reached this country in this connection, and we must all trust that there has been no occasion for them.

### BOOTS FOR THE BELGIAN WOUNDED.

We have the pleasure to acknowledge the receipt of 15s. 6d. from Miss Ethel Laurence for one pair of boots. Four new pairs of Army Blücher boots have been dispatched to the General Hospital, Tunbridge Wells, for four wounded soldiers at that hospital, where the men arrived in a deplorable condition, new boots being a real pressing want of most of them. We shall be glad to supply further boots where they are needed, if the secretary of the hospital where the wounded men requiring such boots may be lying will write to us, giving the sizes and any further particulars as to the date when the boots should be delivered.

### "THE GLAMORGAN AND MONMOUTH HOSPITAL FOR THE FRENCH ARMY."

Those interested in the scheme for the above-named institution, which was noted in The Hospital of November 28 (page 191), will like to know that the amount received towards its equipment is now over £4,000. Mrs. Marcus Paterson, of Cardiff, recently journeyed to France to make certain arrangements, and there interviewed Colonel Sir Almroth Wright, Consulting Physician to the British Expeditionary Forces, and General Bery, Surgeon-General of the Army in Northern France. A fully furnished hotel, fitted with electric light and central heating, and situated at a health resort on the French coast, has been accepted. The building has accommodation for 100 or more beds, and it has been decided to increase the number accordingly.

### HOMES FOR INCURABLES AND THE WAR.

When the supporters of the Royal Hospital for Incurables at Putney met to celebrate Founders' Day, Mr. Thomas W. Wickham, L.C.C., the chairman of the board of management, said they had been asked what they were going to do for sailors and soldiers who had been incapacitated through serving their country. In the cases of those past medical help, the board would make investigation, and, so far as they were able, would make a home for them. Among those associated with the hospital who have joined the Colours are Lord Wolverton, the president; Captain T. Clarence Goff is with the Royal Scots, Mr. W. Allcroft has joined the Royal Fusiliers, and Mr. J. Truscott the Inns of Court Officers' Corps. A son of the steward, Mr. John Miller, has been wounded, but happily is now convalescent. A report had been circulated that in consequence of the war several necessaries which those in the hospital had been in the habit of receiving had been stopped; but Mr. Wickham was able to say that the board had been unable to find that anything had been stopped, and that so long as the public allowed them, there would be no alterations.

### HONORARY STAFFS AND HOSPITAL BOARDS.

Dr. Pratt, the senior physician of the Leicester Royal Infirmary, made an interesting point at the recent meeting of the board. In supporting the resolution regretting Sir Edward Wood's resignation of the chairmanship, Dr. Pratt paid him the tribute of being, in his opinion, the only hospital chairman to place the whole of the members of the honorary medical staff on the board, in addition to giving them very substantial representation on all the committees of the institution. Further, the speaker continued, Sir Edward had given representation to the members of the local branch of the British Medical Association, who now sent two members to the board. In ways like this Sir

Edward Wood had always legislated, and had invariably obtained the entire approval of the honorary staff and the medical profession generally in the work which had been undertaken.

### MEDICAL MEN AS EAGER COMMITTEEMEN.

These remarks are worth quoting because, on general grounds, they point to the fact, so invariably insisted on in The Hospital during twenty-five years, that where difficulty arises between the honorary staff and a hospital, or between the local medical profession and a hospital, it is invariably due to the absence of a proper, regular, and free channel of intercommunication between the two. That is why the adequate and generous representation of the medical profession on hospital boards is so essential. But there is this also to be said. From the administrative side, from the side of the board and the secretary, the complaint is made that medical men, even members of honorary staffs, will not take advantage of the representation offered to them, and, as the phrase goes, "only turn up at committee meetings when something of immediate importance to them happens to be at stake." May we not, therefore, in genuine admiration gild the laurels which Dr. Pratt has rightly placed upon Sir Edward Wood's reputation as a great hospital chairman by this tribute from the administrative point of view, that one of his titles to fame is that he has induced the honorary staff and the local profession to seek and enjoy every representative right held out to them?

### FROM SECRETARY AND HOUSE SURGEON TO DISPENSER AND SECRETARY.

Except in the case of a medical superintendent, the division of labour and division of interest between the medical and administrative staffs is fairly broad, and therefore interest attaches to the one hospital, if recollection serves, which has combined the offices of secretary and house surgeon. This is the Aberystwyth Infirmary and Cardiganshire General Hospital, which has now decided to divide the two offices that have hitherto been held by Dr. Davies and his successor, Dr. Jones. At a special meeting of governors lately held it was decided to appoint a secretary of the infirmary, and the salary offered was £15 per annum and 5 per cent. on all subscriptions and collections, donations being excluded. The institution possesses twenty-eight beds, and the latest figures available give its annual income as £747. On these figures the total salary would work out apparently at £50 (£15 + £35), or possibly less, according to the respective part played by collections or donations in the annual income. It is interesting to note that Dr. Edward Roberts, Penywern, the chairman, announced that eight applications had been received for the post. Of these the short list, after one withdrawal, amounted to four; three candidates were men and one a woman. The latter was eventually appointed, and Miss Myfanwy Jones is therefore the first secretary. It should be added that Miss Jones already holds the post of dispenser at the infirmary, and it is not apparent whether or not the tradition of the institution to combine some other office, as in the past that of house surgeon, with the position of secretary is to be discontinued. Presumably the recent decision to divide the house surgeon's and secretary's posts must have been come to not merely on grounds of circumstance, but because of a feeling that two such different offices should, as a matter of principle, be held by different officials. The change from secretary and house surgeon to dispenser and secretary seems no easier to justify in principle, and it would be very interesting to hear what rule of theory or convenience of practice has led the governors of the Aberystwyth Infirmary to act in this apparently empirical way.

### £10,000 FOR THE WALSALL HOSPITAL.

Under what the lay Press would describe as romantic circumstances the Walsall and District Hospital is to benefit by the sum of £10,000 under the will of the late Mr. James Mason, of Melbourne, Australia. Dying at the advanced age of ninety-four, a Walsall man by birth, the testator, it is curious to note, left his native town some years before the Walsall Hospital was founded, in 1863, and he never returned to see the institution which he has benefited so signally. It is also curious to read the terms of the bequest, which are contained in the following clause in his will, that the Mayor of Walsall communicated to the hospital's chairman, Mr. F. J. Cotterell, who made them public at a meeting of the executive committee last week. The clause reads: "I direct my executors to remit to the Mayor and Councillors of the Corporation of Walsall, Staffordshire, England, the sum of ten thousand pounds, with which to erect a James Mason Ward at the site or adjoining the present hospital, and for the support and upkeep of the same in all and any necessary expense to carry out the object of such an institution as the Walsall Hospital." Such a clause reveals what Englishmen are apt to forget, or even to regard as an amiable fiction—namely, the custom of Colonials, whether English-born or not, to refer to England simply and naturally as "home." That is the feeling, we take it, which prompted this bequest.

### THE NEW MASTER IN LUNACY.

The announcement is made that Sir David Brynmor Jones has been appointed a Master in Lunacy, with the result that a Parliamentary vacancy occurs in Swansea District, which constituency has been represented by the new Master for some twenty years, since 1895 in fact. In passing, we cannot help wondering whether the election thus caused will create much political interest; at all events, it will test practically the reality of the professions that were made that all political controversy should be suspended at this crisis.

### THE SPIRIT OF THE R.A.M.C.

The spirit animating the medical officers during their work in the field, and a vivid sense of the dangers to which they are subject, is given in a remarkable letter signed E. B. Jardine, Lieut.

R.A.M.C., 21st Field Ambulance, 7th Division, which has been published in the *Daily Mail.* "I observe," says the writer, "in your edition of November 24, in a column entitled 'How the British stuck it at Ypres,' a reference to myself. I should like to point out that the gallant conduct attributed to me in that article is entirely erroneous. The mistake arises from the writer confusing me—not for the first time—with my brother-officer Lieutenant W. Ingram. He was later attached to the Royal Scots Fusiliers when their gallant medical officer, Captain Dickson, was wounded." After having thus gallantly taken the newspaper to task for its mistake, Lieutenant Jardine proceeds: "No praise can be too great for the medical officers and their stretcher-bearers attached to regiments, who were subject not only to continuous shell fire in the course of their work, but also to the deadly sniping on their hazardous journeys to and from the trenches. No individual praise could be elicited from me after having seen so many brave men face death with a smile . . . or the medical officer behind them, calmly bandaging men in some farm under heavy shell fire with but one thin British line in front. There was a time before Ypres when men knew that it was merely a question of time and their names would be added to the 'Roll of Honour.' They faced it cheerfully—and the line is still held." How well, we may again remind our readers, does this emphasise the peculiar risks and kind of courage demanded of medical officers at the Front, on which our own medical contributor with the Forces has insisted.

### MEDICAL MEN AS PRISONERS OF WAR.

THE statement now published that the French protest, made through the Spanish Ambassador in Berlin against the violation by Germany of the rules of the Geneva Convention respecting doctors, pharmacists, and medical service men when taken prisoners, has been successful, is to be noted. The result is, then, that Germany undertakes to send back to France some of the medical service men who had been detained in the Fatherland. The retaliatory detention by France of similar medical *personnel* has also stopped. For the ethics of this question and the practical absurdity on either side of a detention policy our readers may be referred to the leading article on the subject which we published on November 7.

### A DEVONPORT HOSPITAL AND MUNICIPAL AID.

THE financial condition of the Royal Albert Hospital, Devonport, is giving rise to anxiety among the members of the board, on the ground that, had not over £3,000 stock been sold out, the hospital could not have paid its way. At the recent annual meeting, presided over by the Bishop of Exeter in his capacity of President, the question of municipal aid was raised. The Bishop recalled with satisfaction the terms offered last year by the Devonport Corporation that they would make a substantial contribution if they were represented on the committee by three members. Mr. Foster J. Bone, in appealing for increased annual subscriptions, said that he did not like accepting contributions from municipal authorities, for hospitals were better carried on voluntarily. We quite agree; but as there seems to be some confusion in the mind of the Bishop and Mr. Bone as to the right and wrong way of securing municipal aid for voluntary hospitals, we would remind them of a leading article which appeared in THE HOSPITAL of June 27 last, in which, discussing "grants in aid," we said: "The voluntary hospitals, despite the heavy additional burden cast on them" by the Insurance Act, "have weathered the storm and proved that they can carry on the work with increased efficiency providing . . . that power is given to the municipalities throughout the country, subject to the sanction of the Treasury or Local Government Board, to make grants in aid as occasion may arise . . . grants in aid which have been recognised by Parliament already in connection with voluntary hospitals, notably in the case of Liverpool (Royal Infirmary)." If the secretary, Captain C. W. Dickinson (or to give him his correct title, honorary secretary), were to press his inquiries on these lines, we have little doubt that the committee of the Royal Albert Hospital, Devonport, would be in a position fully to realise what terms they ought to accept from the municipality.

### THE WAR AND COUNTRY PRACTICE.

To those who watch the advertisement columns of the chief medical journals week by week it becomes apparent how hardly pushed some institutions are for resident medical officers. After several weeks of fruitless advertising at the usual salary an increase is offered, to be followed in a couple of weeks by a further increase. In one instance of late the salary has been raised from £100 to £200, and yet the advertisement still appears. On a smaller scale a similar scarcity occurred during the South African War, and the salaries of *locum tenentes* rose very considerably. The present difficulties are, perhaps, likely to become more acute when arrangements are being made to provide a medical service for the battalions of the new Army, which will doubtless at no distant date be warned for service abroad. Country practitioners who are called will suffer the most, for those in towns are able to arrange for their practices to be carried on by colleagues, but in sparsely populated districts the difficulty cannot be so easily overcome in this way. Such practitioners should surely have a prior claim to temporary assistance. The general point to notice is, however, that men will not be willing again, as in the past, to accept the old low salaries offered.

### THE TASK OF THE BRITISH RED CROSS SOCIETY.

As those who read our leading article last week realise we have nothing but praise for the work which the British Red Cross Society is doing, and obviously this work has meant substantial financial support and help from many people. But, rightly or wrongly, there is an impression abroad that the Council is a somewhat arbitrary organisation, and that its authority is to a large extent a self-determined one. Thus there has arisen in certain quarters a resolution to keep clear from the Society's control and to take an individual and independent course. In times like the present we recognise this to be unfortunate, but we cannot say that, in the circumstances, it is altogether unexpected. Enthusiasm and anxiety to help may be guided, but they will not be driven, and in these democratic days those who provide the moneys required must be given some voice in their control. If there is to be universal zeal, attention must be given to local wishes, and even to local prejudices; and there must be at least the semblance of local management. The task of the British Red Cross Society is to secure these in such a fashion that all shall be directed to one and the same end, and until it has done this there will exist in greater or less measure the tendencies which have been deplored so recently.

### THE CENTRAL COUNCIL FOR DISTRICT NURSING.

The inaugural meeting of the Central Council for District Nursing in London was held on December 1, 1914, in the Conference Hall of the Local Government Board. It will be recalled that the Council has been established as the result of two previous conferences, at which it had been resolved that "a Central Council for London should be established consisting of representatives of those engaged in district nursing and of others interested in the question, with the object of so systematising the arrangements for district nursing that there shall be adequate provision throughout the county." Mr. Samuel, in receiving the Council, said that the advent of the war had a profound influence upon the question of organising an adequate nursing service. The demand for nurses was greater than ever owing to the military need, but when the war was over there would be a larger body than before of women—women who will have had experience and training. Sir William J. Collins, K.C.V.O., proposed by the Rev. J. Scott Lidgett, D.D., was elected as chairman, and Dr. Christopher Addison, M.P., proposed by J. Arthur Dawes, Esq., M.P., seconded by Frank Briant, Esq., J.P., as vice-chairman of the Council. On taking the chair, Sir William Collins said that his experience in St. Thomas's Hospital and in the mean streets of Hatton Garden and Cloth Fair had taught him the desperate need for qualified district nurses, and he gathered that it was desired not to supersede or supplant existing organisations, but to supplement, support, and perhaps subsidise the benevolent operations of voluntary agencies. An executive committee of twenty-five members was then appointed, and after its terms of reference had been decided on and its members elected, votes of thanks concluded the proceedings.

### THE SUCCESS OF A CENTRAL DRUG STORES.

The Lambeth Board of Guardians have had before them the results of the working of their new scheme for outdoor dispensing for the poor. When they decided to establish a central drug stores at their infirmary they concluded that they might with advantage close a dispensary at Montford House, Kennington, an outdoor relief station where medicines were dispensed for the outdoor poor. With the sanction of the Local Government Board they entered into agreements with a number of chemists in the borough to dispense prescriptions for those living some distance from the infirmary on the same terms as those prevailing under the National Insurance Act. Three months have elapsed since the inauguration of the experiment, and the clerk was at the close able to report to the Guardians that, whereas the accounts of the chemists for the quarter amounted to £32 2s. 10d., the average cost in the preceding two years, for the same period, of drugs and salaries of dispensers was approximately £65 a quarter. The former figures included all establishment charges, but the latter did not. There is little wonder that the Guardians expressed their gratification at the result of their experiment, which was very reluctantly sanctioned by the Local Government Board, and then only for twelve months. It is the intention of the Board in the future to extend the experiment, and so dispense with another dispensary in their borough.

### THE QUESTION OF CHRISTMAS FESTIVITIES.

Our readers will have noticed with interest the decision of the London Hospital, which was recorded in our last issue, to cancel all Christmas festivities, mainly because of the double duties now falling to most of the resident medical men, and to the exceptionally heavy demands now made on the lay staff. The possible arrival of a contingent of wounded on Christmas Day itself had also to be considered. We dwell upon these facts because there seems to be a feeling of uncertainty in some quarters as to what is the right policy to pursue. We think that the above, however, provides the answer. The amount of Christmas festivities must be conditioned by the circumstances of each particular institution. Thus, in the case of children's hospitals every effort is generally being made to continue Christmas rejoicings as usual, and no doubt a similar policy, so far as possible, will be pursued by the smaller hospitals also. The decision must depend on what work, if any, is being done for the soldiers, and also on the numbers and duties of the hospital staff at this moment of emergency. Facts of this sort will make the direction which the final decision should take fairly obvious, but we believe that, when once the facts have been bowed to, there will be a wish, so far as possible, to keep the spirit, if not the letter, of Christmas festivities this year on the old lines.

### THE LONDON AMBULANCE SERVICE.

While it does not now seem likely that the London County Council's ambulance service will be in full swing by the end of the year, the latest news from the special committee of the Council is that three ambulance stations will be fully equipped and a partial service from these stations in operation before the end of the financial year, which occurs in March. It is also hoped that the remaining three stations will be completed early in the next financial year. The estimates already approved amount to £5,495, and the committee have been asked to prepare at an early date a comprehensive estimate of the annual cost of the service.

### EFFECT OF THE WAR ON THE DRUG SUPPLY.

The first evening meeting of the Pharmaceutical Society's present session was held at 17 Bloomsbury Square, London, on December 8, when several papers were read regarding the effect of the war on the nation's drug supply. Professor H. G. Greenish dealt with the question of crude drugs, and expressed the opinion that the number of medicinal plants at present under cultivation in different parts of the British Empire might be very considerably increased; he also said we had to ask ourselves whether it was not possible to find a substitute for a drug, the supply of which had been seriously curtailed as a result of the war; necessity might compel us to search for parallel drugs, and, in the event of such not being forthcoming, to prosecute the cultivation of the drug we required in one of the divisions of the Empire, and so render ourselves less dependent upon sources of supply that might at such times as the present fail altogether. Mr. J. C. Umney said the action of the Government in connection with a Drug Supply Advisory Committee in framing an effective schedule of contraband of war had done much to prevent clearance of existing stocks, and so protected the interests of our land and sea forces as well as those of the civil population. The action of the Government, by their inquiries and legislative power of acquisition, had levelled prices from the range of fancy to fact in the short space of two or three weeks.

### THE MANUFACTURE OF SYNTHETIC DRUGS.

On the occasion of the above meeting, Mr. C. A. Hill, referring to the manufacture of synthetic drugs, said we could not expect to build up in a few months an industry which had taken forty years to develop in Germany. One of the great difficulties was that each product depended on others, the raw materials of one synthetic substance being a by-product in the manufacture of another. It would not pay to put down expensive plant for the manufacture of a few products, the whole consumption of which was only a matter of perhaps hundredweights, but it would pay a factory which could turn out a hundred products each with a consumption of some hundredweights per annum. Mr. R. R. Bennett dealt with the effects of the patent law on the drug supply. Mr. F. W. Gamble reviewed the position with regard to serums and vaccines; since the outbreak of war large quantities of typhoid vaccines had been supplied to the War Office, and there had been a heavy demand for calf lymph, antisepsis vaccine, and tetanus antitoxin. We quote this discussion as showing how alive are pharmacists to the difficulties of the problems which they are invited to solve, and at the same time how willing they are, with what assistance may be necessary, to do what they can under the circumstances. We can only hope that the lesson of what should be done, taught by the war, will not be forgotten when peace once more is proclaimed.

### THE LEAGUE OF MERCY ANNUAL MEETING.

The annual meeting of Presidents of the League of Mercy and the presentation of the Order of Mercy will take place at 3 o'clock on Thursday, December 17, at St. James's Palace. Princess Alexander of Teck will make the presentation and Lord Farquhar will be in the chair.

### THE SARDONIC PORTER.

In the brilliant article on Hospital Finance, which we lately published, two points were made. The first was the readiness with which the educated public listens to tales told them by out-patients as to the treatment meted out to them; and the second dealt, as The Hospital has often done, with the value of a discreet, courteous, sympathetic gate porter, and his value in enlisting, or turning away, support from the hospital's doors. Well! Since the above article appeared we have heard an out-patient story, which we tell for what it is worth as illustrating our contributor's contentions. An out-patient visited a certain eye hospital, and found himself, as is generally the case, somewhat at the mercy of the porter, a man of immense appearance and, as it proved, sardonic humour. "What is that dog making all that noise for?" inquired one of the patients. "We keep that dog," replied the porter, "to eat the eyes taken out of the patients." No comment is needed. As a stupid joke" it will never be forgotten; imagined, or real, it will be remembered against the institution rather than the man.

### THIS WEEK'S DRUG MARKET.

A fair business is passing, and while prices are, generally speaking, steady, there have been several noteworthy exceptions. Owing to the very heavy demand for resublimed iodine the price is substantially higher; tincture of iodine is being used by all the armies in the field for the treatment of wounds, and while there appears to be no scarcity of the drug the output is abnormally large. All the soldiers in the French Army are provided with a little ampoule containing a weak aqueous solution of iodine with potassium iodide, and there appears to be a movement on foot to supply the British Army with something similar. Ipecacuanha is substantially dearer as a result of a large demand occasioned by the increased use of emetine. There is little change in the position of opium and its alkaloids, but a further advance in price is by no means improbable. The salicylates have an upward tendency in price. Atropine continues scarce and dear. Liquid paraffin still has an advancing tendency. Potassium chlorate is again dearer. Cod-liver oil is quiet, but quotations have a tendency to advance. Among the articles which have a lower tendency are citric acid, cream of tartar, potassium bromide, sodium bromide, and essence of lemon. Oil of peppermint and eucalyptus oil are offered at comparatively low rates.

